# Difference in lesion formation by male and female *Pratylenchus penetrans*

**DOI:** 10.21307/jofnem-2020-090

**Published:** 2020-08-25

**Authors:** Kanan Saikai, Ann E. MacGuidwin

**Affiliations:** Plant Pathology Department, University of Wisconsin–Madison, 491 Russell Laboratories, 1630 Linden Dr., Madison, WI, 53706

**Keywords:** Alfalfa, Dill, Lesion nematode, Pea, Symptoms

## Abstract

*Pratylenchus penetrans* induce necrotic lesions, the hallmark symptom for the genus, soon after infection. The objective of our study was to characterize and quantify gender differences in lesion development. Independent experiments were conducted *in vitro* for three hosts; pea (*Pisum sativum* L. cv. Early Alaskan), dill (*Anethum graveolens* cv. Long Island Mammoth), and alfalfa (*Medicago sativa* cv. Vernal). Each experimental unit was an excised radical placed on water agar in a Petri dish and inoculated with either 40 adult males or 40 fourth-stage juvenile females. Length, size, and number of lesions were recorded during the experiment and the radicals were harvested 14 days after introducing nematodes. Lesions were first observed on pea after two days for female-inoculated roots, and 24 hr after introducing both genders to dill and alfalfa. Lesions expanded either by multiple lesions coalescing or individual lesions expanding over time. Males made fewer, smaller lesions with less discoloration for all three hosts. There was no difference among genders for the total number of nematodes recovered per Petri dish or the number of endoparasitic nematodes after 14 days. The survival rate of males and females at harvest was not different, indicating that the difference in lesion formation was not related to nematode population densities. This study verified and quantified the observation that lesions induced by males are less extensive and in smaller numbers than lesions by females.

*Pratylenchus penetrans* is an economically important species with a wide geographic distribution in temperate climates. Unlike some other species in the genus, *P. penetrans* requires females and males for reproduction and, therefore, males are common (Thistlethwayte, 1970; Mamiya, 1971). The gender ratio varies, depending on habitat, hosts, and soil conditions (Patterson and Bergeson, 1967). Both genders are migratory throughout the life cycle, feeding on the root surface as an ectoparasite or in the root cortex as an endoparasite on a broad range of hosts (Zunke, 1990a). The common name of root-lesion nematode describes its hallmark symptom of tissue necrosis in roots.

The formation of lesions is closely associated with migration and nematode feeding (Townshend et al., 1989; Zunke, 1990b). Infection by *P. penetrans* occurs along all regions of roots and the nematode migrates within roots inter- and intracellularly, resulting in minute lesions (Townshend and Stobbs, 1981). The nematode migrates through cells by breaking down successive cell walls, and these injured cells lose membrane integrity and cell organelles degenerate (Townshend et al., 1989). Lesions coalesce and the intensity of discoloration increases with time (Townshend and Stobbs, 1981). Zunke (1990b) detailed the infection and feeding behavior of *P. penetrans* on rape, oil radish, tobacco, and potato as five phases; probing, cell penetration by the stylet, salivation, brief, and extended feedings. Shortly after feeding commenced, light brown longitudinal discoloration was induced across a few cells, which later became larger lesions harboring multiple females and eggs (Zunke, 1990b). The secretion of saliva has been documented during feeding and is considered to be the source of effector proteins, such as cell wall degrading enzymes, known to be produced by *P. penetrans* (Fosu-Nyarko and Jones, 2016; Vieira et al., 2018).

Previous studies using single-stage inoculum of *P. penetrans* found that males preferred to remain outside roots as compared to females. Males were less likely than females to penetrate roots of alfalfa (Sontirat and Chapman, 1970; Townshend, 1978; Olthof, 1982) and more likely to exit pea roots occupied by the same gender (Wixted and MacGuidwin, 1990). In a study using mixed inoculum, Oyekan et al. (1972) reported that males were more likely to exit lesioned areas in pea roots, resulting in a 5:1 infection ratio of females to males. Townshend (1978) demonstrated that female *P. penetrans* penetrated roots at a broader range of temperature than males. Wixted and MacGuidwin (1990) observed roots occupied only by males had less apparent discoloration than roots with females, but no study tested the hypothesis that males and females were equal for the ability to induce lesions.

Our goal was to confirm gender differences in lesion formation for *P. penetrans.* Clarifying potential bias related to the gender ratio of inoculum benefits research to understand root damage inflicted by *P. penetrans* and the relationship of lesions to root function. The objectives of our study were to compare the dynamics, number, and size of lesions formed by male and female *P. penetrans.* We studied pea and alfalfa, the plants used in the original observations about gender differences in infectivity (Sontirat and Chapman, 1970; Townshend, 1978; Olthof, 1982) and egress (Oyekan et al., 1972; Wixted and MacGuidwin, 1990) and dill to verify that gender differences occurred for more than one plant family.

## Materials and methods

Three separate experiments using three hosts and a common design were conducted for 14 days at 24°C. Pea (*Pisum sativum* L. cv. Early Alaskan), dill (*Anethum graveolens* cv. Long Island Mammoth), and alfalfa (*Medicago sativa* cv. Vernal) axenic root explants were inoculated with male or female nematodes using a randomized complete block design with gender and time main effects and four blocks (replications) representing the sequence of inoculation and data collection, and position of the Petri dishes during incubation. The experiment was repeated twice (dill and alfalfa) or three times (pea).

### Seed sterilization

Pea seeds were surface sterilized by soaking in 95% ethanol for 5 min and 10% NaOCl for 20 min, followed by two sterile water rinses. Dill and alfalfa seeds were soaked in 70% ethanol for 2 min, and 2% NaOCl for 3 min, followed by three sterile water rinses. After incubating in the dark for 4 to 5 days at room temperature, one radical cut to 3-cm for pea and 2-cm for dill and alfalfa, was transferred to a Petri dish containing 1.2% water agar (WA). Petri dishes were held for 3 days at 24°C prior to the introduction of nematodes so discolored radicals could be culled.

### Nematode introduction to roots

Sterile nematodes were collected from 3-month old monoxenic cultures of *P. penetrans* reared on ‘IO Chief’ sweet corn root explants. Roots were incubated in sterile water for 24 hr to allow nematode egress. Forty fourth-stage female juveniles or 40 adult males were hand-picked from the nematode inoculum into 1-ml sterile water. Using sexually immature females eliminated the possibility of reproduction occurring during the experiment. The final molt to the adult stage occurred during the 14-day experiment in all cases, as females were no longer juveniles at day 7 on dill and alfalfa. Nematodes were placed 0.5 cm away from a root tip. A minimum of three root explants of each host were also reared in Petri dishes without nematodes as a negative control for lesion development.

### Data collection

The number of lesions was counted at the same time every day and the length of individual lesions was measured using a Leica MZFLIII stereomicroscope (Leica Microsystems Ltd, Heerburgg, Switzerland) with the CellSens Software (Olympus Corporation, Tokyo, Japan). The area of each lesion was measured at seven and 14 days after introducing nematodes for dill and alfalfa using NIS-Elements AR Software on a Nikon Eclipse Ti-E inverted microscope (Nikon Instruments Inc, Melville, NY). Nematode behavior was also video-recorded using NIS-Elements AR Software on at least one randomly selected nematode per Petri dish for 15 min at days 7 and 14 on dill and alfalfa.

The number of nematodes in a root (endoparasites) as well as the number remaining in the Petri dish was counted on day 14. Pea roots were cleared and stained with acid-fuchsin (Byrd et al., 1983) to count nematodes. Dill and alfalfa roots were transparent and thin enough to mount directly into a drop of water. The number of nematodes observed on the outer root surface prior to removing the root was counted as ectoparasites. The nematodes that were more than 0.5 cm away from roots were counted as egressed. The average number of nematodes per lesion and per mm^2^ of lesion was calculated. Counts of endoparasites were confirmed for the dill and alfalfa experiments by dissecting roots, and the viability of nematodes was assessed by activity and turgor pressure upon rupturing the nematode cuticle. Survival rate was computed by dividing the (number of live nematodes) by the (number of total nematodes) observed × 100. Pea roots were stained to count endoparasites, which killed nematodes so no survival rate was computed.

### Statistical analysis

Statistical analyses for lesion data were conducted separately for each experiment and response variable using a mixed model (PROC MIXED, SAS Institute, Cary, NC). Studentized residual plots were generated by PLOTS = STUDENTPANEL. Kenward–Roger method was selected (DDFM = KR) to adjust the degrees of freedom according to the covariance matrix. Gender, time, and the gender × time interaction were included as fixed effects in models for the lesion data with experimental trial, replication nested within trial, and the interaction of gender, trial and replication (whole-plot error) as random effects. Time was treated as a repeated measure. First order autoregressive covariance structure (ar(1) option) was selected because all the data were collected at equally spaced time points. The effect of gender was compared at each time point by performing a partitioned analysis of the least square means by day for the interaction with the SLICE option and *P-*value adjusted by the Tukey procedure.

The gender effect on the nematode count data at day 14 (number of total nematodes per Petri dish, nematodes per lesion or per mm^2^, endoparasites, ectoparasites, egressed nematodes, and survival rate) were analyzed using generalized linear mixed model (PROC GLIMMIX) for each host. Models included the nematode count data and gender as the response and explanatory variables, respectively, with the random effects of trial and replication nested within each experimental trial. The distribution of each response variable was selected after comparing studentized residual plots (PLOTS = STUDENTPANEL) for preliminary model runs using multiple distributions.

## Results

The total number of nematodes recovered per Petri dish and the number of endoparasites were the same for males and females on all three hosts, as were survival rates on dill and alfalfa (Table 1). Up to 25% of the nematodes were unaccounted for on dill and alfalfa, and 38% for pea. The majority of nematodes were inside roots, but a few were feeding ectoparasitically at harvest and some were egressed. We found a greater (*P* < 0.01) number of nematodes per lesion on pea and alfalfa roots with males (Table 1). The distribution of the nematode demographic data in Table 1 were not normal and fit either the negative-binomial distribution (endoparasite, ectoparasite, and egressed nematodes) or the lognormal distribution (number of nematodes per lesion and survival rate).

**Table 1.** Mean and range of nematode data on roots of pea, dill, and alfalfa inoculated with 40 females or 40 males.

	Pea		Dill		Alfalfa	
	Female	Male	Female	Male	Female	Male
Total nematodes/Petri dish	25 (14-32)	25 (15-35)	35 (29-39)	32 (28-36)	30 (26-35)	31 (26-34)
Average nematode/lesion	1.1 (0.6-1.9)	2.5** (1.6-4.2)	2.7 (2.0-4.7)	2.6 (1.0-4.3)	1.5 (1.0-2.2)	3.3** (1.7-4.6)
Endoparasites	24 (14-32)	22 (15-13)	31 (24-36)	27 (22-33)	27 (24-32)	28 (23-33)
Ectoparasites	–	–	0.6 (0-1)	1.8* (0-6)	0.5 (0-2)	1.1 (0-2)
Egressed nematodes	0.7 (0-4)	2.7* (0-12)	3.8 (2-6)	5.3 (1-12)	3.9 (1-10)	3.1 (1-6)
Survival rate (%)	–	–	97	96	100	94

**Notes:** Means are the average of three trials for pea and two trials for dill and alfalfa. Survival rate and ectoparasites were not measured for pea. Asterisks represent statistically significant differences between female and male. *P < 0.05; **P < 0.01.

### Lesion formation

**Figure 1:**
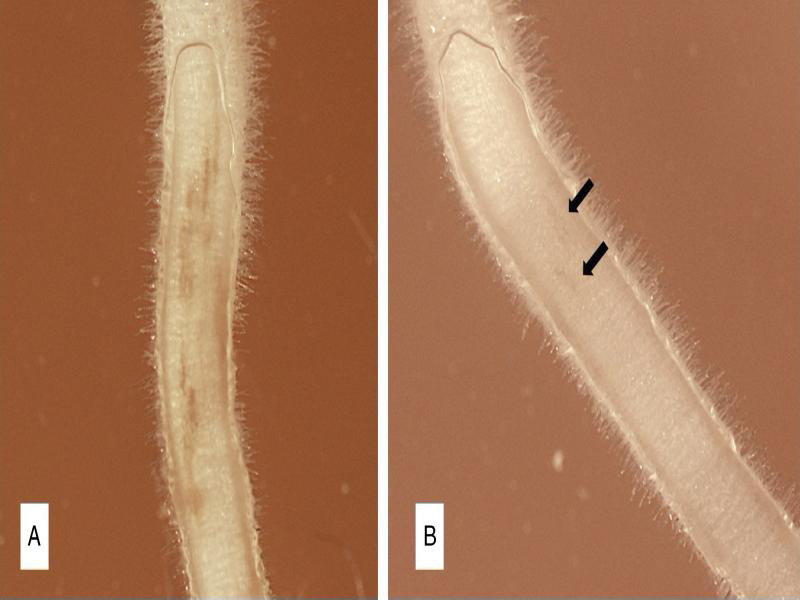
Lesions formed by *Pratylenchus penetrans* females (A) and males (B) on root explants of pea at 14 days after the introduction of the nematodes. The arrow points at lesions on the male-inoculated root.

Lesions were visible on dill and alfalfa one day after inoculation and on pea after two days for both genders. Lesions induced by females first appeared as light brown water-soaked areas on the root surface and became larger, darker, and eventually necrotic for all three hosts. Lesions made by males were more discrete and retained a water-soaked to very light brown appearance throughout the study (Fig. 1). Necrosis was confined to where nematodes appeared to be feeding for both genders. Nematodes were not always found within small lesions, but larger lesions always harbored multiple nematodes. Although some cells in the lesion appeared to lose membrane integrity, the majority of cells in necrotic areas were still intact without ruptured cell walls. Only cells within lesions had a granular appearance. There was limited root growth due to use of WA for the experiment, so root variability and growth was confirmed by transferring control roots to Gamborg’s B5 media at the end of the experiment.

**Figure 2:**
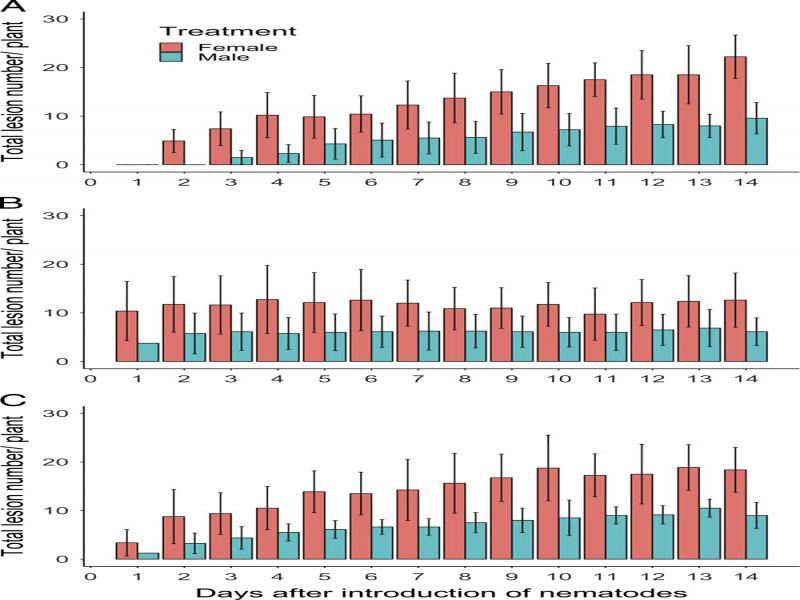
Relationship between time and the number of lesions per plant for roots of pea (A), dill (B), and alfalfa (C) inoculated with 40 *Pratylenchus penetrans* males or 40 *P. penetrans* females. Data bars represent the mean ± SE of three trials for pea and two trials for dill and alfalfa.

Gender (*P* < 0.001 to *P* = 0.03) and time (*P* < 0.001) main effects were significant in repeated measure models for the number of lesions, with females making more lesions than males on all three hosts (Fig. 2). The interaction between the main effects was significant (*P* < 0.001) on pea and alfalfa, but not on dill. On pea, the number of lesions peaked at 22 lesions on day 14 for females and at 7 lesions on day 9 for males. The number of lesions peaked after one day for both genders on dill. On alfalfa, the number of lesions peaked at 14 lesions on day 7 for females and at 4 lesions for males.

**Figure 3:**
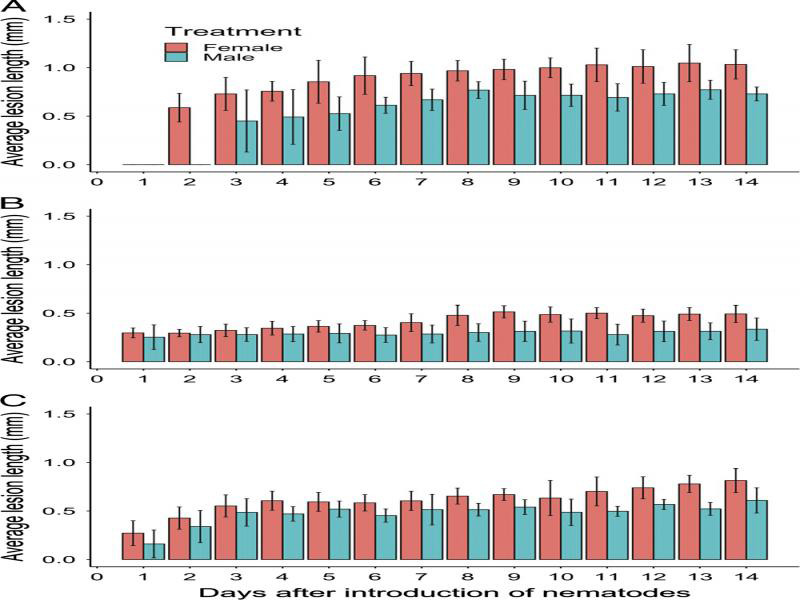
Relationship between time and the average lesion length (mm) for roots of pea (A), dill (B), and alfalfa (C) inoculated with 40 *Pratylenchus penetrans* males or 40 *P. penetrans* females. Data bars represent the mean ± SE of three trials for pea and two trials for dill and alfalfa.

Both gender (*P* < 0.001 to *P =* 0.05) and time (*P* < 0.001) main effects were significant for the average lesion length on the three hosts with a significant (*P* < 0.001) interaction between gender and time for dill (Fig. 3). For females, the average lesion length peaked at 0.92 mm on day 6 for pea, and at 0.60 mm and 0.40 mm on day 7 for dill and alfalfa, respectively. For males, the average lesion length peaked at 0.61 mm on day 6 for pea, at 0.28 mm on day 3 for dill, and at 0.34 mm on day 2 for alfalfa.

**Figure 4:**
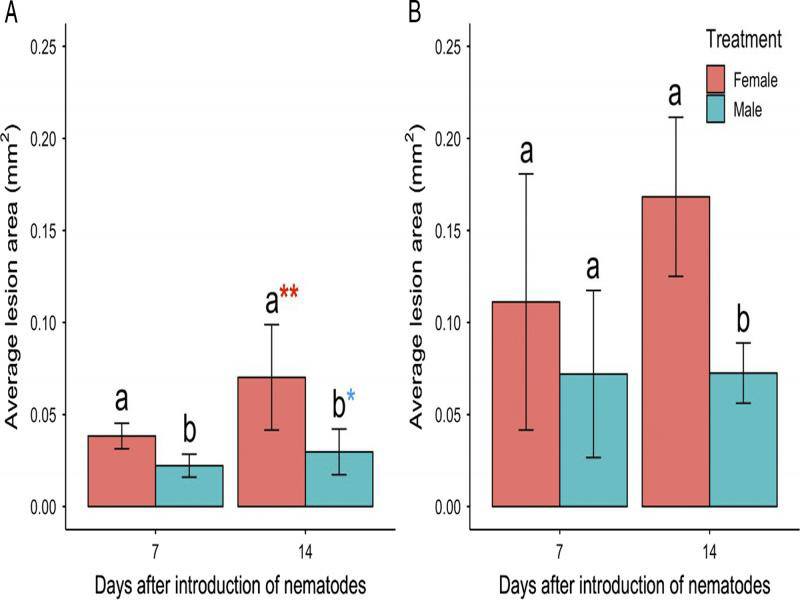
Relationship between time and the average lesion area (mm^2^) for roots of dill (A) and alfalfa (B) inoculated with 40 *Pratylenchus penetrans* males or 40 *P. penetrans* females. Data bars represent the mean ± SE of two trials. Letters represent statistically significant differences between female and male on each day (*P*< 0 .05). Asterisks denote difference between time points at day 7 and day 14.

The average lesion area was greater for females than males at both time points for dill but only on day 14 for alfalfa (Fig. 4). The lesion area continued to increase after lesion length peaked on dill, reaching a maximum on day 14 for both genders. The average lesion area peaked at day 7 for both genders on alfalfa, closer to the time of maximum lesion length.

### Nematode behavior

The majority of nematodes moved toward the root immediately after inoculation and were observed inside roots within 24 hr. Females were more widely distributed along the roots and tended to be less aggregated than males. Males preferred to aggregate at the root elongation zone on alfalfa, but not on dill and pea. The majority of males were close to the cut end of dill roots but did not show this behavior for the other two hosts. No nematodes of either gender were observed feeding on root hairs. It was difficult to visualize nematodes inside pea roots during the experiment. Dill and alfalfa roots were more transparent, but even so not all nematodes were visible through the Petri dish. Pumping activity of the metacorpus was confirmed for both genders on dill and alfalfa.

Movement of both genders was restricted to the cortex on all three hosts. Intercellular migration for both genders was observed on dill and alfalfa based on the video-records of nematode migration and did not result in immediate necrosis. Established lesions were briefly fed upon by females as they passed by. Though most eventually moved into roots, ectoparasitic males often rubbed the surface of pea and dill roots with their head and moved along the roots during the first seven days. The majority of the nematodes inside roots were mostly straight and oriented longitudinally to the root axis. Some nematodes curled up within cells on pea but not on alfalfa or dill.

## Discussion

Male *P. penetrans* made fewer, smaller lesions than females on members of the plant families Fabaceae and Apiaceae, confirming earlier observational reports (Oyekan et al., 1972; Townshend, 1978; Wixted and MacGuidwin, 1990). Lesions made by males were more discrete with less coloration than those made by females. These unambiguous differences were revealed because of our unique design of separating the genders. Root occupancy was the same for males and females, diminishing the possibility that our findings were biased by gender differences in infection competency. We could not account for all of the nematodes placed in Petri dishes, but the discrepancy between the number added and the number recovered was similar for males and females.

Both genders of *P. penetrans* induced lesions within 24 to 72 hr on all three hosts. Lesions first appeared in less than 24 hr for peach (Mountain and Patrick, 1959), cabbage (Acedo and Rohde, 1971), and strawberry (Kurppa and Vrain, 1985), but took longer for some hosts such as lily at 12 days (Vieira et al., 2017) and apple at one month (Pitcher et al., 1960). The necrotic area on roots expanded by either merging lesions or enlarging individual lesions, or both. Lesion appearance was gender-specific and similar for all the three hosts, though symptom progression was slower for dill. Host differences in lesion development have been documented by others *in vitro* (Townshend and Stobbs, 1981) as well as *in planta* (Miller, 1978). Contrary to pea and alfalfa, the host status of dill for *P. penetrans* is not well known except that Miller (1978) documented severe necrosis on dill compared to other hosts. The broad host range of *P. penetrans* includes some members of the Apiaceae, such as carrot (Jensen, 1953), so it would be interesting to evaluate dill and other family members to identify crops with potential to suppress reproduction by this important species.

Migration and feeding behaviors of *P. penetrans* have been linked to the development of symptoms (Zunke, 1990b). Our data indicate that feeding was more important, and perhaps essential, to lesion formation. Females made new lesions on pea for eight days past the day when the average lesion length stopped increasing. Intercellular movement of *P. penetrans* was observed in videos, yet the lesions were discrete and surrounded by apparently healthy tissue. For both genders, lesions corresponded to where nematodes were located at the time of observation rather than the route they had taken to get there. It is possible that our *in vitro* system influenced the relationship between lesion formation and nematode movement, as we did not see intracellular migration as was reported for other hosts (Townshend, 1963a; Townshend and Stobbs, 1981). On the other hand, nematode feeding was evident for both genders in our experiment based on pumping movements of the metacorpus as well as stylet thrusting. We also observed that the intestine of nematodes at day 14 was rarely depleted, indicating nematodes were not starved during experiments. Whereas necrosis was observed at the nematode feeding sites and the nearby intact cells for both genders, the greater number of affected cells and lower number of nematodes per lesion were always associated with females. Our interpretation is that feeding by *P. penetrans*, not migration, was the primary cause of symptom development.

Our observation was that both genders of nematodes were restricted to the root cortex and did not invade endodermal cells or vascular tissues on the three hosts. The endodermis was a barrier to the invasion of the vascular tissues by *P. penetrans* (Townshend, 1963a, 1963b; Vieira et al., 2017; Zunke, 1990b), except for a few cases where damage was noted (Townshend, 1963a; Acedo and Rohde, 1971). Feeding on the endodermis was not observed in the two weeks of two studies (Acedo and Rohde, 1971; Kurppa and Vrain, 1985) so the shorter duration of our study may account for the lack of damage to the endodermis. Unlike previous reports of feeding on root hairs on rape, oil radish, tobacco and potato (Zunke, 1990a), we did not observe root hair feeding on the three hosts for the 14-day period, agreeing with Kurppa and Vrain (1985). Only males appeared to have a preferred region for root penetration.

We were unable to discern cellular responses to the nematode damage. Some of the previously reported reactions of cells to *P. penetrans* feeding (Mai et al., 1977; Zunke, 1990a), such as cell death, ruptured cell walls, and hypertrophied nuclei, were not observed on dill and alfalfa. A small number of cells in each necrotic area showed a disintegrated granular cytoplasm, but the majority of cell walls were intact. Studies refining gender differences in terms of histopathology, effector proteins, and plant defenses would be interesting in light of the advances that are being made in these areas for mixed populations of *P*. *penetrans* (Uehara et al., 2001; Mitreva et al., 2004; Vieira et al., 2015, 2017, 2018, 2019). A recent study showed no difference among male and female *P. coffeae* for expression of two genes that encode plant cell wall degrading enzymes (Bell et al., 2019), but it is not known if the gender differences in lesion formation that we observed for *P*. *penetrans* is also true for *P*. *coffeae*.

In conclusion, *P. penetrans* males induced fewer, smaller, and less distinct lesions than females on three hosts. Aggregation behavior displayed by males within lesions did not compensate for the gender differences. There was less disparity among males and females for the time to first appearance of lesions than for the time required to reach maximum lesion length.

## References

[ref001] Acedo, J. R. and Rohde, R. A. 1971. Histochemical root pathology of *Brassica oleracea capitate* L. infected by *Pratylenchus penetrans* (Cobb) Filipjev and Schuurmans Stekhoven (Nematoda: Tylenchidae). Journal of Nematology 3:62–68. 19322342 PMC2619850

[ref002] Bell, C. A. , Lilley, C. J. , McCarthy, J. , Atkins, H. J. and Urwin, P. E. 2019. Plant-parasitic nemtaodes respond to root exudate signals with host-specific gene expression patterns. PLoS Pathogen 15:e1007503, doi: 10.1371/journal.ppat.1007503. 30707749 PMC6373980

[ref003] Byrd, D. W. , Kirkpatrick, T. and Barker, K. R. 1983. An improved technique for clearing and staining plant tissues for detection of nematodes. Journal of Nematology 15:142–143. 19295781 PMC2618249

[ref004] Fosu-Nyarko, J. and Jones, M. G. K. 2016. Advances in understanding the molecular mechanisms of root lesion nematode host interactions. Annual Review of Phytopathology 54:253–278. 10.1146/annurev-phyto-080615-100257 27296144

[ref006] Jensen, H. J. 1953. Experimental greenhouse host range studies of two root-lesion nematodes, *Pratylenchus vulnus* and *Pratylenchus penetrans*. Plant Disease Reporter 37:384–387.

[ref007] Kurppa, S. and Vrain, T. C. 1985. Penetration and feeding behavior of *Pratylenchus penetrans* in strawberry roots. Revue Nématology 8:273–276.

[ref009] Mai, W. F. , Bloom, J. R. and Chen, T. A. 1977. Biology and ecology of the plant parasitic nematode *Pratylenchus penetrans*. Research Bulletin 815 The Pennsylvania State University College of Agriculture, Pennsylvania.

[ref008] Mamiya, Y. 1971. Effect of temperature on the life cycle of *Pratylenchus penetrans* on Cryptomeria seedlings and observations on its reproduction. Nematologica 17:82–92.

[ref010] Miller, P. M. 1978. Reproduction, penetration, and pathogenicity of *Pratylenchus penetrans* on tobacco, vegetables, and cover crops. Phytopathology 68:1502–1504.

[ref011] Mitreva, M. , Elling, A. A. , Dante, M. , Kloek, A. P. , Kalyanaraman, A. , Aluru, S. , Clifton, S. W. , Bird, D. , McK., Baum, T. J. and McCarter, J. P. 2004. A survey of SL1-spliced transcripts from the root-lesion nematode *Pratylenchus penetrans*. Molecular Genetics and Genomics 272:138–148. 15338281 10.1007/s00438-004-1054-0

[ref012] Mountain, W. B. and Patrick, Z. A. 1959. The peach replant problem in Ontario VII. The pathogenicity of *Pratylenchus penetrans* (Cobb, 1917) Filip. & Stek. 19411. Canadian Journal of Botany 37:459–470.

[ref013] Olthof, T. H. A. 1982. Effect of age of alfalfa root on penetration by *Pratylenchus penetrans*. Journal of Nematology 14:100–105. 19295681 PMC2618150

[ref014] Oyekan, P. O. , Blake, C. D. and Mitchell, J. E. 1972. Histopathology of pea roots axenically infected by *Pratylenchus penetrans*. Journal of Nematology 4:32–35. 19319243 PMC2619918

[ref015] Patterson, S. M. T. and Bergeson, G. B. 1967. Influence of temperature, photoperiod, and nutrition on reproduction, male-female-juvenile ratio, and root to soil migration of *Pratylenchus penetrates*. Plant Disease Reporter 51:78–82.

[ref016] Pitcher, R. S. , Patrick, Z. A. and Mountain, W. B. 1960. Studies on the host-parasite relations of *Pratylenchus penetrans* (Cobb) to apple seedlings. I. Pathogenicity under sterile conditions. Nematologica 5:309–314.

[ref017] Sontirat, S. and Chapman, R. A. 1970. Penetration of alfalfa roots by different stages of *Pratylenchus penetrans* (Cobb). Journal of Nematology 2:270–271. 19322310 PMC2618750

[ref018] Thistlethwayte, B. 1970. Reproduction of *Pratylenchus penetrans* (Nematoda: Tylenchida). Journal of Nematology 2:101–105. 19322280 PMC2618722

[ref019] Townshend, J. L. 1963a. The pathogenicity of *Pratylenchus penetrans* to strawberry. Canadian Journal of Plant Science 43:75–78.

[ref020] Townshend, J. L. 1963b. The pathogenicity of *Pratylenchus penetrans* to celery. Canadian Journal of Plant Science 43:70–74.

[ref021] Townshend, J. L. 1978. Infectivity of *Pratylenchus penetrans* on alfalfa. Journal of Nematology 10:318–323. 19305861 PMC2617914

[ref022] Townshend, J. L. and Stobbs, L. 1981. Histopathology and histochemistry of lesions caused by *Pratylenchus penetrans* in roots of forage legumes. Canadian Journal of Plant Pathology 3:123–128.

[ref023] Townshend, J. L. , Stobbs, L. and Carter, R. 1989. Ultrastructural pathogenicity of cells affected by *Pratylenchus penetrans* in alfalfa roots. Journal of Nematology 21:530–539. 19287649 PMC2618970

[ref024] Uehara, T. , Kushida, A. and Momota, Y. 2001. PCR-based cloning of two β-1,4-endoglucanases from the root-lesion nematode *Pratylenchus penetrans*. Nematology 3:335–341.

[ref025] Vieira, P. , Mowery, J. , Eisenback, J. D. , Shao, J. and Nemchinov, L. G. 2019. Cellular and transcriptional responses of resistant and susceptible cultivars of alfalfa to the root lesion nematode, *Pratylenchus penetrans*. Frontiers in Plant Science 10:971, doi: 10.3389/fpls.2019.00971. 31417588 PMC6685140

[ref026] Vieira, P. , Mowery, J. , Kilcrease, J. , Eisenback, J. D. and Kamo, K. 2017. Characterization of *Lilium longiflorum* cv. ‘Nellie White’ infection with root-lesion nematode *Pratylenchus penetrans* by bright-field and transmission electron microscopy. Journal of Nematology 49:2–11. 28512372 10.21307/jofnem-2017-040 PMC5411250

[ref027] Vieira, P. , Eves-van den Akker, S. , Verma, R. , Wantoch, S. , Eisenback, J. D. and Kamo, K. 2015. The *Pratylenchus penetrans* transcriptome as a source for the development of alternative control strategies: mining for putative genes involved in parasitism and evaluation of *in planta* RNAi. PLoS ONE 10:e0144674, doi: 10.1371/journal.pone.0144674. 26658731 PMC4684371

[ref028] Vieira, P. , Maier, T. R. , Eves-Van Den Akker, S. , Howe, D. K. , Zasada, I. , Baum, T. J. , Eisenback, J. D. and Kamo, K. 2018. Identification of candidate effector genes of *Pratylenchus penetrans*. Molecular Plant Pathology 19:1887–1907. 10.1111/mpp.12666 PMC6638058 29424950

[ref029] Wixted, D. J. and MacGuidwin, A. E. 1990. Differences in egress of male and female *Pratylenchus penetrans* from pea roots. Journal of Nematology 22:614–617. 19287768 PMC2619089

[ref030] Zunke, U. 1990a. Ectoparasitic feeding behavior of the root lesion nematode, *Pratylenchus penetrans*, on root hairs of different host plants. Revue de Nématologie 13:331–337.

[ref031] Zunke, U. 1990b. Observations on the invasion and endoparasitic behavior of the root lesion nematode *Pratylenchus penetrans*. Journal of Nematology 22:309–320. 19287726 PMC2619059

